# Should e-cigarette use be included in indoor smoking bans?

**DOI:** 10.2471/BLT.16.186536

**Published:** 2017-04-28

**Authors:** Nick Wilson, Janet Hoek, George Thomson, Richard Edwards

**Affiliations:** a Department of Public Health, University of Otago, Mein St, PO Box 7343, Wellington, 6021, New Zealand.; b Department of Marketing, University of Otago, Dunedin, New Zealand.

Electronic nicotine delivery systems, also called e-cigarettes, are devices that vapourize liquid, typically comprising nicotine, propylene glycol, glycerine and flavourings. Switching from smoking tobacco cigarettes to using e-cigarettes – known as vaping – may reduce user harm, by supporting quitting or acting as a lower risk substitute. However, the degree of harm reduction is uncertain. Governments that are considering policies to restrict vaping should consider the optimal regulation of e-cigarette products, including defining where vaping may occur. Here, we explore some of the arguments for and against extending indoor smoke-free laws to also cover vaping.

## Arguments for vaping

First, allowing vaping in indoor public places may encourage smokers to switch to vaping, by making it relatively more attractive as vaping would be allowed where tobacco smoking is not. Some e-cigarette users have voiced this potential benefit of normalization of vaping when arguing against any bans on public vaping.^1^ Nevertheless, we are not aware of any clear evidence supporting this argument as an important driver for smokers switching to vaping. Other factors, such as health reasons or the lower cost of vaping, seem to be more important for switching from smoking to vaping. Furthermore, if vaping indoors does actually normalize vaping for smokers, then logic would suggest it might also normalize vaping for non-smokers.

Second, allowing vaping in indoor public places where smoking is not permitted could minimize any discomfort that e-cigarette users may experience from nicotine withdrawal when being in such settings. However, evidence suggests that this discomfort is fairly modest. For example, in a survey conducted among exclusive e-cigarette users in the United States of America, only 12% (124 of 1034) reported finding it difficult to refrain from vaping in places where they were not supposed to.^2^

## Arguments for prohibiting vaping

First, at a distance, smoking and vaping may look similar to some people, since both activities produce visible clouds exhaled from people’s mouths after they have drawn on a cigarette or device. Some e-cigarette users admit to this similarity, e.g. some cite visual similarity as a reason why they do not vape around people who are eating.^1^ Given such similarities, permitting indoor vaping might renormalize tobacco smoking in smoke-free indoor environments and may lead smokers to query: if vaping is permitted, why is smoking not allowed. Renormalization of tobacco smoking would be particularly problematic if it increases the risk that children become susceptible to or initiate smoking. Indeed, some research suggests that children may misperceive vaping as smoking.^3^ Nevertheless, the authors of this study speculated that “once these products are more common and the purpose of them is known, seeing people use them should normalize quitting behaviour.”^3^

A second argument is that close exposure to vaping among people who have recently quit smoking or vaping might trigger them to relapse to smoking. For example, an experimental study among young-adult tobacco smokers reported that exposure to a video showing vaping significantly increased their urge to smoke as well as their desire for tobacco cigarettes and e-cigarettes.^4^ Similarly, another experimental study found that exposure to the e-cigarette cue but not the tobacco cigarette cue also significantly increased desire to smoke an e-cigarette.^5^

Evidence suggests that many smokers support smoke-free areas, because this helps encourage them to quit.^6^ It seems plausible that this reasoning would also apply to e-cigarette users, who wish to either constrain the level of their vaping or to quit vaping and may therefore favour indoor areas being vape-free.

Third, passive exposure to e-cigarette vapour might lead to adverse health effects according to a systematic review of 16 studies.^7^ A 2016 report from the World Health Organization (WHO)^8^ also concluded that second-hand aerosols from e-cigarettes are a new air contamination source for hazardous particulate matter (PM). The levels of some metals, such as nickel and chromium, in second-hand aerosols are not only higher than background air, but also higher than second-hand smoke. Furthermore, compared to background air levels, PM_1.0_ and PM_2.5_ in second-hand aerosols are 14–40 times and 6–86 times higher, respectively. In addition, nicotine in second-hand aerosols has been found to be between 10–115 times higher than in background air levels, acetaldehyde between two and eight times higher, and formaldehyde about 20% higher.^8^ The report suggested that the increased concentration of toxicants from second-hand aerosols over background levels poses an increased risk for the health of all bystanders, especially those with pre-existing respiratory conditions.^8^

As a result of the report, WHO recommends to Parties of the Framework Convention on Tobacco Control (FCTC) that they consider prohibiting by law the use of e-cigarettes in indoor spaces or at least where smoking is not permitted.^8^ Furthermore, the International Agency for Research on Cancer^9^ now considers particulates such as PM_2.5_ to be carcinogenic. These data seem to support the case for fairly strong precautionary arguments for governments to protect the public from involuntary exposure to second-hand aerosols.

Fourth, regardless of the potential health risks, some people find second-hand aerosols from nearby vaping to be a nuisance, since the e-cigarettes can include strong flavours and leave pungent odours. While such nuisance concerns do not appear to have been quantified in surveys, we note that the 2016 vaper-friendly Global Forum on Nicotine conference, actually banned participants from vaping in certain indoor areas due to the nuisance that aerosol clouds caused.^10^

Fifth, a law aiming to achieve high compliance needs to be readily understandable to people who vape and those around them, hence a law restricting smoking should support a smoke-free encompasses vape-free approach. Exemptions that permit vaping in some indoor smoke-free settings (e.g. certain workplaces, restaurants or pubs) but not others, may risk generating confusion. The problems with a lack of simplicity have been illustrated by jurisdictions that have adopted complex smoke-free laws (e.g. exemptions for some types of small pubs/bars, permitting smoking rooms and defining half an indoor area smoke-free). Simplicity might also favour citizen-led promotion and enforcement of the law by reducing confusion between a cloud of vaped aerosol at a distance and a cloud of cigarette smoke.

## Conclusion

Considering the above arguments collectively, we believe that, from a public health perspective, central and local governments should adopt regulations that effectively determine that all designated indoor smoke-free areas are also vape-free areas. We note that this approach is being implemented by many jurisdictions, with vaping being banned in enclosed public spaces, such as bars, restaurants and other workplaces, in 25 countries.^11^ This approach is also recommended in the 2016 WHO report to the Parties of the FCTC.^8^

Nevertheless, further research on the risks of using e-cigarettes is still desirable.^12^ Research is needed to determine whether smoke-free outdoor areas should also be vape-free or not, as the issues differ somewhat from indoor public spaces (e.g. greater dilution of second-hand aerosols outdoors).

An important perspective is whether a society is considering vaping as a permanently acceptable activity or as a temporary way to provide nicotine for people giving up smoking and transitioning to be nicotine-free. If public health policies are based on the latter perspective, it may be unwise to adopt any policy permitting indoor vaping areas, since that could suggest vaping should be a permanently allowed activity. Furthermore, governments wanting to encourage smokers to shift to vaping might be better advised to evaluate the potential of other strategies, such as differential prices, that is, via high tobacco taxes and untaxed e-cigarettes. A potential advantage of price instruments over vape-free policies is that price instruments might be more easily and quickly adjusted via tax changes than changes to the legal designation of vape-free areas.
